# Hot Deformation Behavior, Constitutive Model, and Processing Map of 022Cr12Ni10MoTi Alloy

**DOI:** 10.3390/ma19194170

**Published:** 2026-09-29

**Authors:** Xiuxuan Yang, Wanhua Chen, Changjun Wang, Jianxiong Liang, Chengguo Yu

**Affiliations:** 1 Facility Design and Instrumentation Institute, China Aerodynamics Research and Development Center, Mianyang 621000, China; 2 Central Iron and Steel Research Insititute, Beijing 100081, China

**Keywords:** 022Cr12Ni10MoTi alloy, hot deformation, microstructure, constitutive model, processing map

## Abstract

This study investigates the hot deformation behavior of 022Cr12Ni10MoTi alloy over a wide range of strain rates and temperatures. Hot compression experiments were carried out at deformation temperatures ranging from 900 °C to 1200 °C and strain rates varying from 0.01 s^−1^ to 10 s^−1^. The microstructure and flow stress of the 022Cr12Ni10MoTi alloy during hot deformation were comprehensively characterized. On the basis of the experimental data, a constitutive model was developed, and processing maps were constructed to delineate the domains of optimal workability. The results demonstrate that the deformation behavior of the 022Cr12Ni10MoTi alloy was significantly influenced by both temperatures and strain rates. Dynamic recovery and recrystallization phenomena were found to occur during hot deformation, which affect the flow stress. The established constitutive model can accurately predict the flow stress of the material during hot deformation. Through the processing maps, the feasible hot deformation regime was identified, enabling the successful fabrication of large-scale alloy forgings.

## 1. Introduction

The 022Cr12Ni10MoTi alloy is a low-temperature martensitic steel that exhibits excellent strength, toughness, and cryogenic performance, making it a promising candidate for aerospace applications, including large-scale cryogenic components and engine valves. Hot forging is a critical manufacturing step for large structural parts, as the elevated temperatures involved profoundly affect the microstructure and mechanical properties of the material. Mandal et al. [1] reported that when the forging temperature of SA508 grade 3 steel was increased from 880 °C to 1046 °C, the flow stress during hot deformation decreased progressively, while the microstructure transformed from relatively coarse martensitic laths to finer ones. Furthermore, the high content of alloying elements, coupled with the multi-factor, non-steady-state, high-temperature, and high-pressure conditions inherent in hot forging, may induce defects in large structural components, such as cracking, microstructural inhomogeneity, and dimensional distortion [2]. These issues severely threaten the service reliability of large pressure-bearing structures and have become a key bottleneck constraining the high-quality and high-efficiency forming of 022Cr12Ni10MoTi alloy large-scale components. Therefore, a thorough investigation into the hot deformation behavior and underlying mechanisms of 022Cr12Ni10MoTi alloy is urgently needed to enable the optimization and precise control of hot forging processes, thereby facilitating the fabrication of large structural components with desirable microstructures and superior mechanical properties.

The mechanical properties and microstructural evolution of stainless steels during hot deformation have been extensively investigated [3,4,5]. It is generally recognized that alloy steels undergo work hardening, dynamic recovery, and dynamic recrystallization during hot deformation [6], with deformation temperature and strain rate exerting significant influences on both flow stress and microstructures [7,8,9]. Li et al. [10] investigated the hot deformation of Ti-(Nb)-Mo steel using a Gleeble-3500 thermomechanical simulator across a range of temperatures and strain rates. Their results revealed that dynamic recrystallization was promoted under conditions of high temperature and low strain rate, whereas pronounced dynamic recovery was exhibited at high strain rates. Increasing the deformation temperature and decreasing the strain rate were observed to enhance the volume fraction of martensite. They also noted that variations in dynamic recrystallization and recovery induced by different thermo-mechanical parameters could lead to marked differences in flow stress. Yang et al. [11] studied the hot deformation of 40Cr10Si2Mo steel, examining the variations in mechanical properties and recrystallization mechanisms. They proposed that the stress–strain curves during hot deformation exhibit three stages: work hardening, flow softening, and steady state. Ansari et al. [12] focused on the hot deformation of M300 martensitic alloy steel, systematically analyzing the evolution of microstructure and its effect on flow stress. In a related study, Qi et al. [13] investigated the hot deformation behavior of 110Mn12Cr2NY alloy and found that the critical strain for dynamic recrystallization decreased with increasing temperature and decreasing strain rate. Considerable variations in microstructure and mechanical properties were observed at different temperatures and strain rates. The optimal processing window was identified as temperatures between 950 and 1200 °C with strain rates ranging from 0.4 s^−1^ to 1 s^−1^. Similar findings have been reported for materials including the Zn-1.2Mg alloy [14], Co-Cr-Mo-C alloy [15], and a medium-entropy superalloy [16].

Constitutive models play a critical role in describing the hot deformation behavior of materials over a wide range of strain rates and temperatures. Several widely recognized models have been developed to characterize flow stress evolution under hot-deformation conditions, including the Arrhenius [17], Johnson-Cook [18], and Zerilli-Armstrong [19] models. Among these, the Arrhenius model is particularly prevalent in hot deformation studies and has been successfully applied to a variety of materials, such as Mg-Gd-Y-Zr [20], P550 steel [21], and high-nitrogen stainless steel [22]. To account for the influence of strain on flow stress, the Zener–Hollomon parameter (*Z*) has been incorporated into the Arrhenius model, which substantially enhances the predictive accuracy of the model during hot deformation [23]. In recent applications, Zhou et al. [24] investigated the hot deformation behavior of 17-4 PH martensitic stainless steel, established a constitutive model, and constructed processing maps to delineate the optimal hot working window. Dourandish et al. [25] examined the mechanical properties and microstructural evolution of martensitic stainless steel during hot deformation; they developed an Arrhenius-type constitutive model and corresponding processing map, the accuracy of which was subsequently validated. Similarly, Zhang et al. [26] established an Arrhenius model and processing map for Fe–21Cr–15Ni–5Mn–2Mo alloy, and further analyzed the underlying mechanisms governing microstructural evolution during hot deformation.

To date, limited research has been devoted to the hot deformation behavior of 022Cr12Ni10MoTi alloy, and a systematic understanding of its microstructural evolution, flow stress, constitutive models, and processing maps under hot working conditions remains lacking. In view of the practical requirements for the hot forming of large-scale 022Cr12Ni10MoTi alloy components, the present study undertakes a comprehensive investigation into its hot deformation characteristics. The mechanical responses and microstructural evolution during hot deformation are systematically characterized, a constitutive model is established, and hot processing maps are constructed to identify the optimal forming windows. On the basis of these studies, hot forging experiments are further conducted on large-scale components.

## 2. Experimental Details

**Figure 1:**
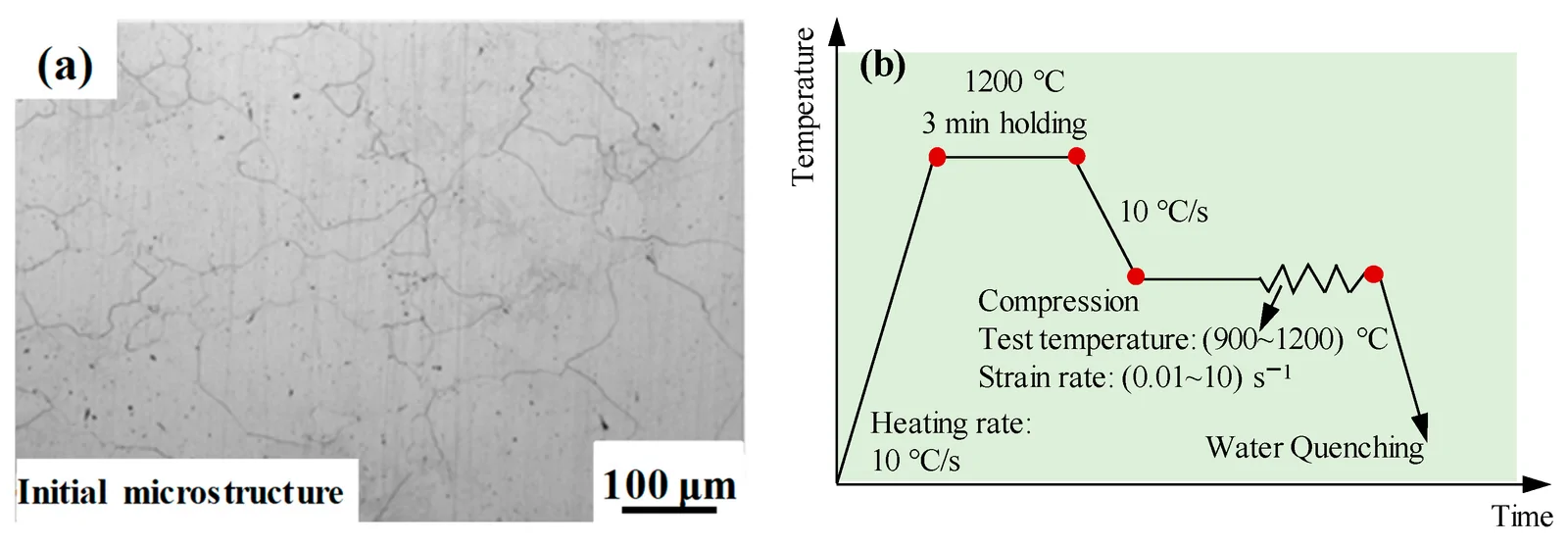
Initial microstructure (**a**) and Heat treatment schematic (**b**) of 022Cr12Ni10MoTi alloy.

The material used in this study is 022Cr12Ni10MoTi alloy, which was produced by vacuum melting. The initial microstructure and chemical composition of the alloy are shown in Figure 1a and Table 1, respectively. It can be seen from Figure 1a that the initial grains are equiaxed with sizes ranging from 200 to 300 μm. The hot compression test was performed on a Gleeble 3800 thermo-mechanical simulator (Dynamic Systems Inc., Poestenkill, NY, USA). The experimental procedure is schematically illustrated in Figure 1b. The samples were machined to a dimension of *Φ*8 × 12 mm with an aspect ratio of 1.5, and a compression height reduction of 60% was applied during testing. Tantalum sheets and graphite foils were placed between the sample ends and the compression anvils to reduce interfacial friction. A thermocouple was welded at the axial midpoint of the sample side surface, achieving a steady-state temperature control accuracy of ±1 °C. The Gleeble tests were conducted under an argon atmosphere to prevent surface oxidation and material loss. The samples were first heated to 1200 °C at a rate of 10 °C/s and held for 3 min to ensure a homogeneous microstructure, then cooled to the target deformation temperature at the same rate of 10 °C/s. The deformation temperatures were selected as 900 °C, 950 °C, 1000 °C, 1050 °C, 1100 °C, 1150 °C and 1200 °C, and the strain rates were 0.01 s^−1^, 0.1 s^−1^, 1 s^−1^, and 10 s^−1^. Upon completion of compression, the samples were immediately water-quenched to preserve the high-temperature microstructures for subsequent analysis. For each experimental conditions, three repeated Gleeble tests were conducted to guarantee the reliability of the experimental results. The deformed samples were ground, mechanically polished, and chemically etched following standard metallographic procedures, after which the microstructure of the specimens was characterized by scanning electron microscopy (SEM, Sigma 300, Carl Zeiss AG, Oberkochen, Germany).

## 3. Experimental Results

**Figure 2:**
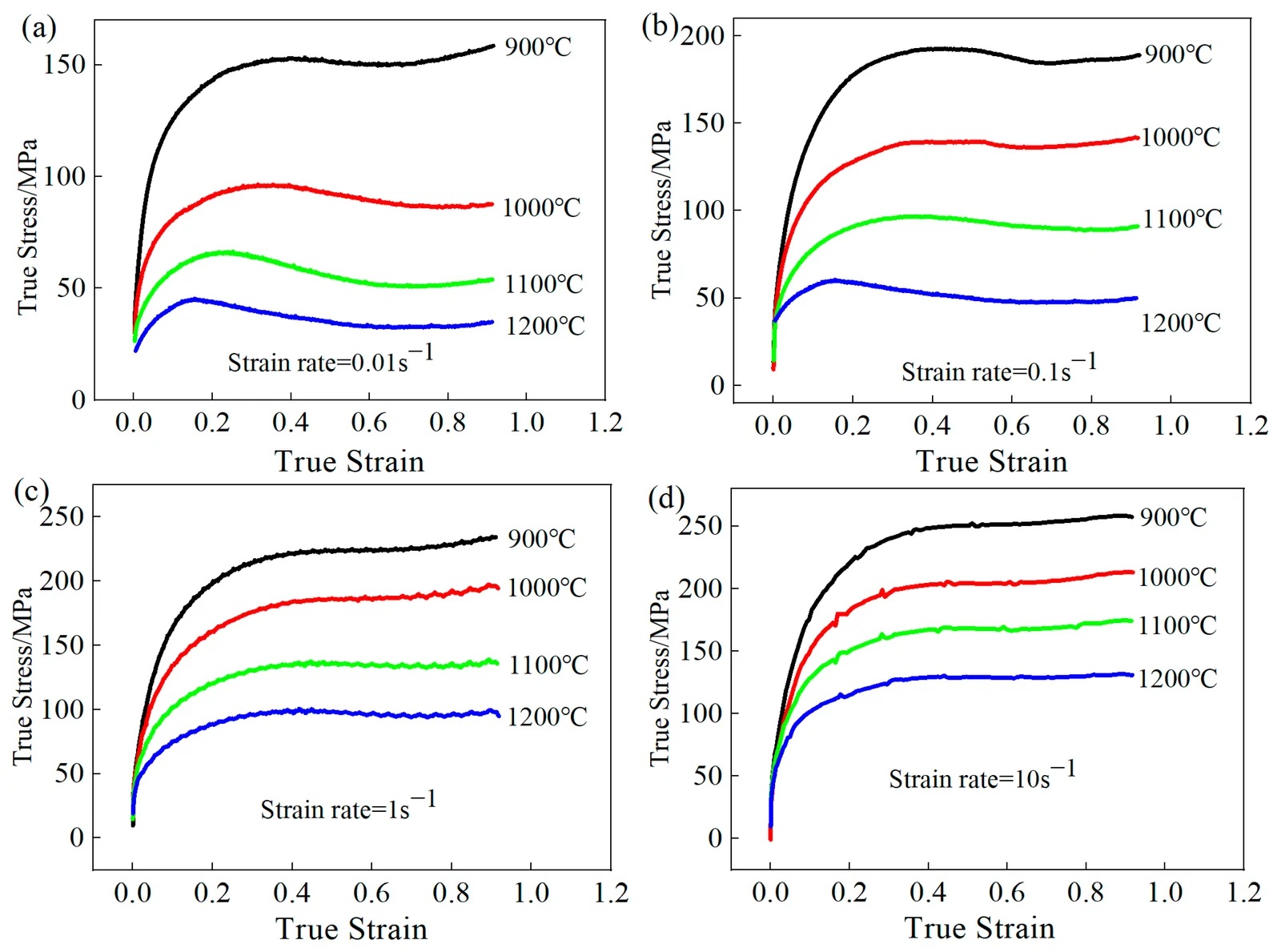
The flow stress of 022Cr12Ni10MoTi alloy under different hot deformation conditions. (**a**) Strain rate = 0.01 s^−1^. (**b**) Strain rate = 0.1 s^−1^. (**c**) Strain rate = 1 s^−1^. (**d**) Strain rate = 10 s^−1^.

Figure 2 presents the stress–strain curves of the 022Cr12Ni10MoTi alloy at various deformation temperatures and strain rates. It can be observed that at the same strain rate, the flow stress of the material decreases with increasing deformation temperature, indicating an obvious thermal softening phenomenon. At a constant deformation temperature, the flow stress rises as the strain rate increases, which represents a notable strain-rate hardening effect.

At a strain rate of 0.01 s^−1^ and temperatures of 1000 °C, 1100 °C and 1200 °C (Figure 2a), the flow stress first increases, then decreases and finally remains stable with rising strain. In contrast, the stress rises at a later stage at 900 °C. This may be because lower temperatures result in less energy input during metal deformation and a weaker softening effect. Severe plastic deformation causes strain hardening to exceed the softening effect induced by recovery and recrystallization, leading to an increase in stress. As the strain rate increases, at a strain rate of 10 s^−1^ and deformation temperature of 900 °C, the flow stress rises gradually and then plateaus with increasing strain. The corresponding stress–true strain curve shows the typical feature of dynamic recovery. Under compression at a high strain rate (i.e., strain rate = 10 s^−1^), the material lacks sufficient time to undergo recrystallization and subsequent softening during compression, so no significant flow stress reduction is observed throughout the entire deformation process. Accordingly, the high-temperature deformation of the 022Cr12Ni10MoTi alloy is governed by the synergistic effects of dynamic recovery and dynamic recrystallization, and these microstructure evolution mechanisms are strongly dependent on temperature and strain rate.

**Figure 3:**
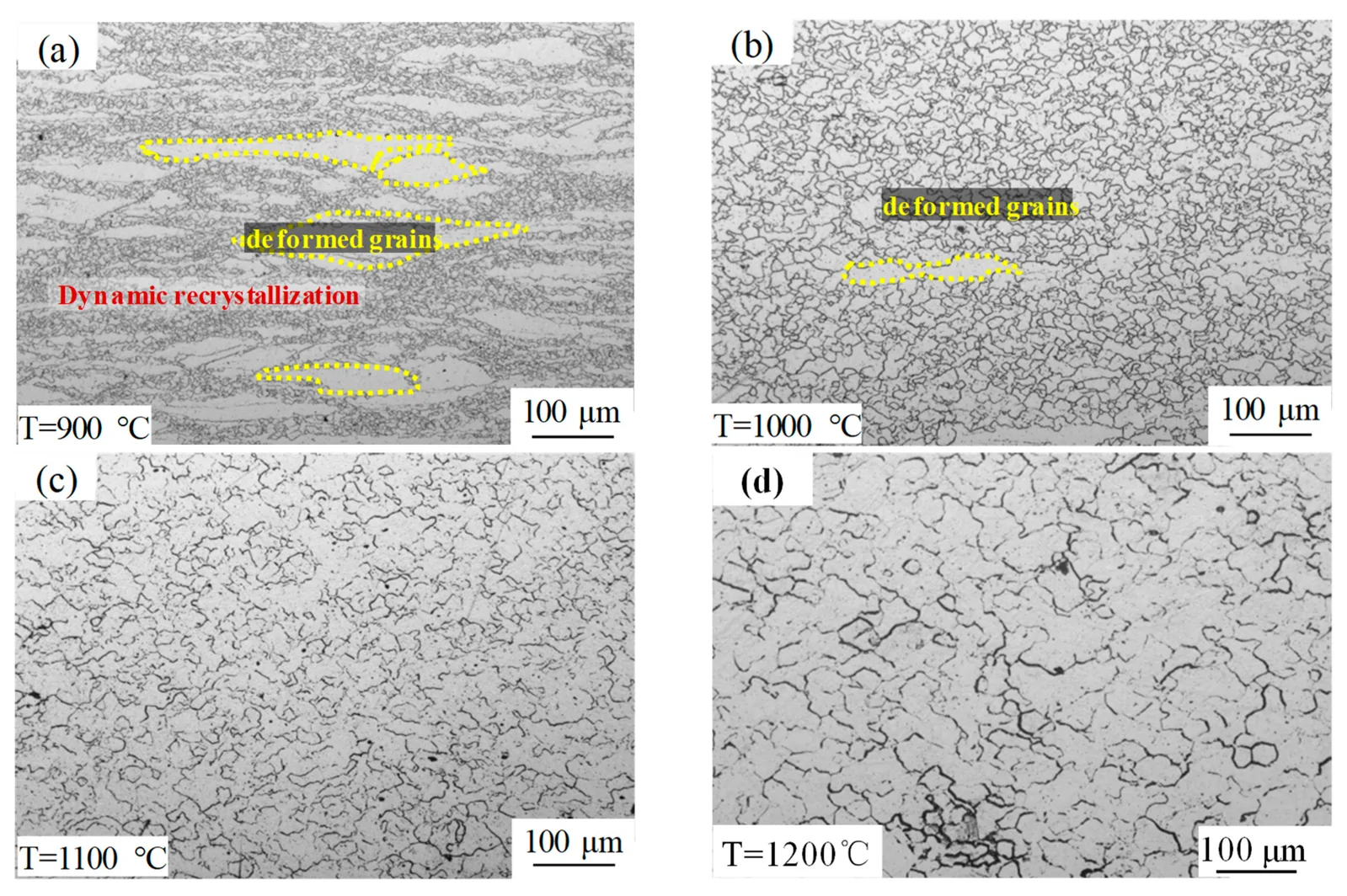
Microstructures of hot deformed 022Cr12Ni10MoTi alloy at 0.01 s^−1^ strain rate and different temperatures. (**a**) T = 900 °C. (**b**) T = 1000 °C. (**c**) T = 1100 °C. (**d**) T = 1200 °C.

Figure 3 presents the SE results of the material deformed at a strain rate of 0.01 s^−1^ and different temperatures. For the 022Cr12Ni10MoTi alloy, the temperature for the martensite-to-austenite reverse transformation is approximately 600 °C, and the transformation finish temperature is about 750 °C. Therefore, in the range of 900–1200 °C, the specimen is predominantly composed of austenite, and no phase transformation occurs during Gleeble tests conducted above 900 °C. At 900 °C (Figure 3a), some elongated large-sized deformed grains can be observed. This is because low temperatures are unfavorable for dynamic recovery and recrystallization of the material. Consequently, partially deformed grains do not undergo recrystallization at this relatively low temperature. The softening effect induced by dynamic recovery and recrystallization is weakened, which increases the stress value. This may account for the rise in flow stress at the later stage at 900 °C (Figure 2a). As the temperature rises to 1000 °C (Figure 3b), the proportion of elongated grains diminishes markedly, while a significant number of fine recrystallized grains emerge throughout the microstructure. At 1100 °C (Figure 3c), the elongated deformed grains disappear completely, and the microstructure becomes predominantly composed of fine, equiaxed recrystallized grains. When the temperature is further increased to 1200 °C (Figure 3d), the recrystallized grains show a noticeable coarsening, indicating that complete recrystallization has occurred during hot deformation, followed by subsequent grain growth under the elevated thermal condition.

## 4. Discussions

### 4.1. Arrhenius Model

Based on the stress–strain curves of 022Cr12Ni10MoTi alloy during hot deformation, a constitutive model describing its high-temperature flow behavior was established. To characterize the interrelationship among flow stress (*σ*), deformation temperature (*T*), and strain rate ($\dot{\varepsilon }$), the constitutive model proposed by Sellars and Tegart [27] was adopted, which is expressed as follows (Equation (1)):

(1) $$\dot{\varepsilon }=\left\{\begin{array}{c} A{\left[\sinh\left(\alpha \sigma \right)\right]}^{n}exp\left(-Q/RT\right)\\ A_{1}\sigma^{n^{\prime }}\left(\alpha \sigma <0.8\right)\\ A_{2}exp\left(\beta \sigma \right)\left(\alpha \sigma >1.2\right) \end{array}\right.$$

where *Q* is the thermal deformation activation energy, *R* is the gas constant with a value of 8.314 J/mol/K [28], and *A*, *n* and α are the strain-dependent material constants, where $\alpha =\beta /n^{\prime }$. By taking the natural logarithm of both sides of Equation (1), the following expression can be obtained:

(2) $$\ln\dot{\varepsilon }=n\ln\left[\sinh\left(\alpha \sigma \right)\right]+\ln A-Q/RT$$

(3) $$\ln\dot{\varepsilon }=n^{\prime }\ln\sigma +\ln A_{1}\left(\alpha \sigma <0.8\right)$$

(4) $$\ln\dot{\varepsilon }=\beta \sigma +\ln A_{2}\left(\alpha \sigma >1.2\right)$$

**Figure 4:**
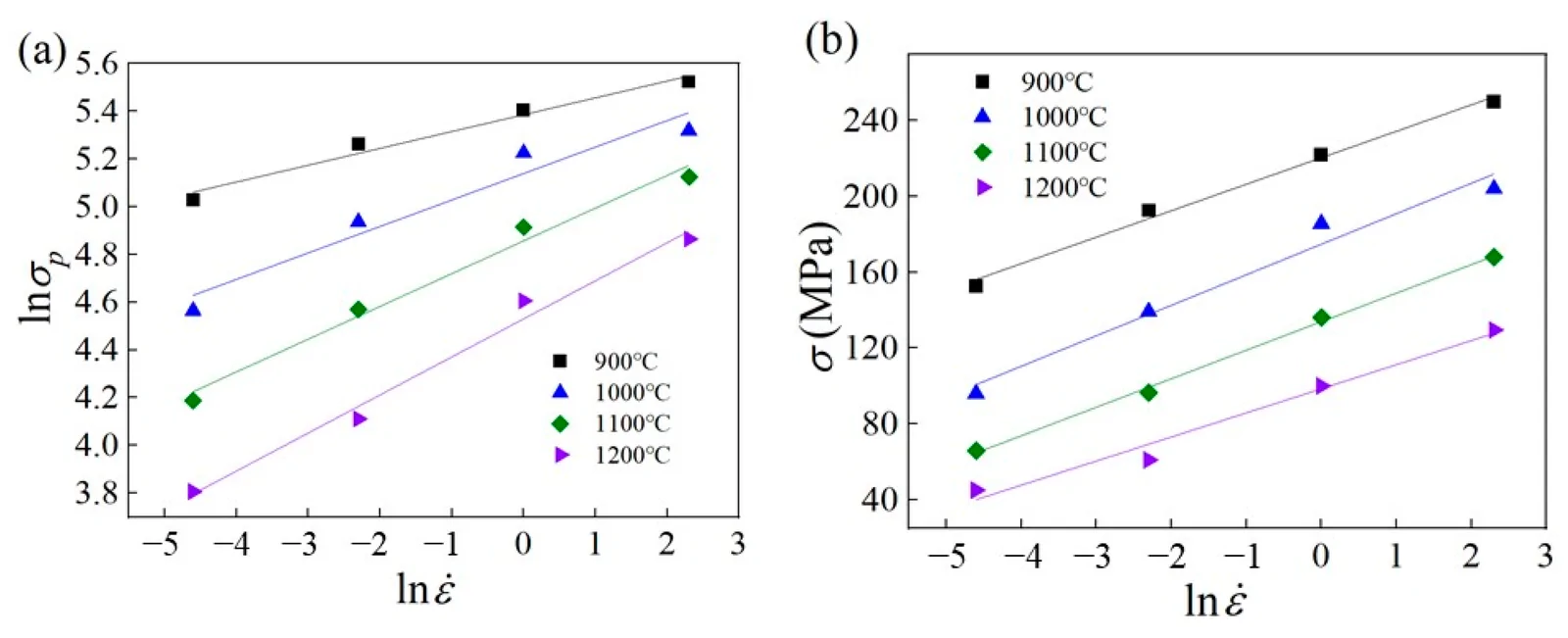
Relationship between stress and strain rate at strain of 0.5. (**a**) Relationship between $\ln\dot{\varepsilon }$ and lnσ. (**b**) Relationship between $\ln\dot{\varepsilon }$ and σ.

The values $n^{\prime }$ and β are determined from the relationship between stress and strain rate. At a strain of 0.5, the relationship between $\ln\sigma -\ln\dot{\varepsilon }$ and $\sigma -\ln\dot{\varepsilon }$ are presented in Figure 4, both of which exhibit approximately linear behavior. From Figure 4a, the value of $n^{\prime }$ was obtained as 8.7704, and from Figure 4b, the value of β was determined as 0.067541. Accordingly, the parameter *α* was calculated as $\alpha =\beta /n^{\prime }=0.0077$.

Taking the partial derivative of Equation (2) with respect to strain rate gives Equation (5):

(5) $$\frac{1}{n}=\frac{\partial \ln\sinh\left(\alpha \sigma \right)}{\partial \ln\dot{\varepsilon }}$$

Similarly, taking the partial derivative of Equation (2) with respect to temperature yields Equation (6):

(6) $$Q=nR\frac{\partial \ln\sinh\left(\alpha \sigma \right)}{\partial \left(1/T\right)}$$

**Figure 5:**
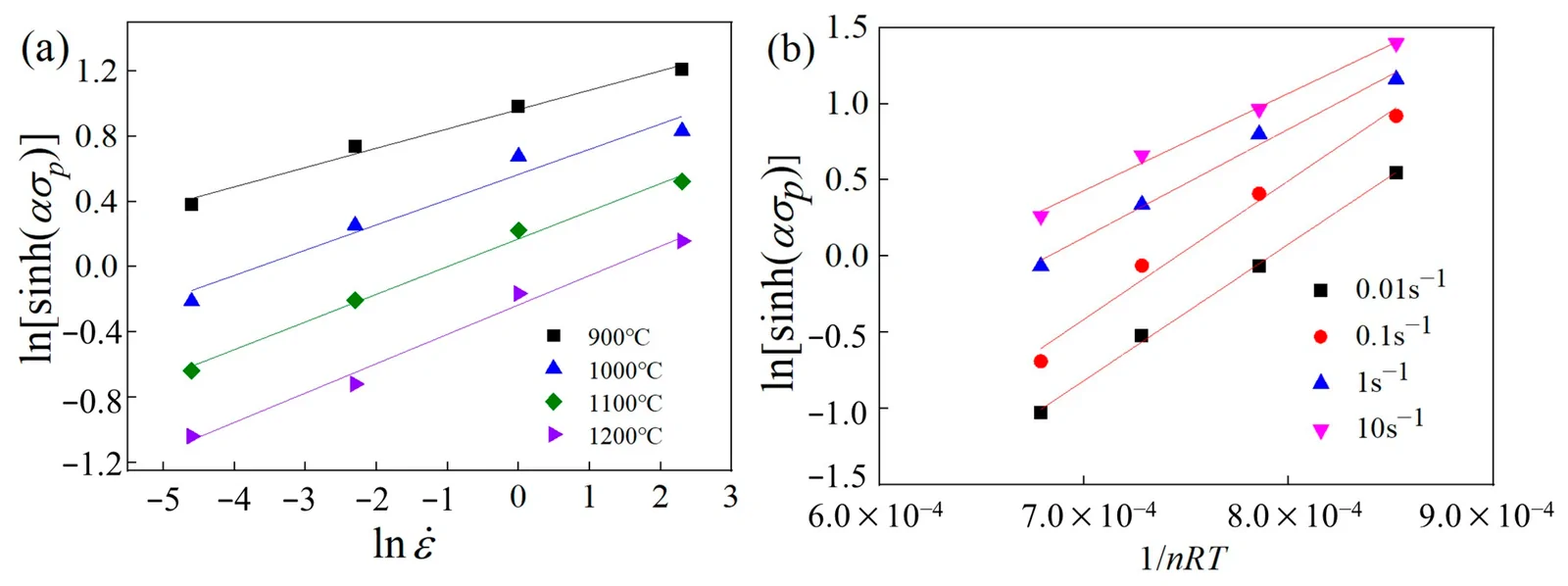
Relationships of flow stress σ with strain rate $\dot{\varepsilon }$ and deformation temperature *T* at strain of 0.5. (**a**) $\ln\dot{\varepsilon }-\ln\left[\sinh\left(\alpha \sigma \right)\right]$. (**b**) $1/T-\ln\left[\sinh\left(\alpha \sigma \right)\right]$.

The *Q* was calculated from the stress σ. The stress σ at strain of 0.5 was adopted for the calculation. The relationships between $\ln\left[\sinh\left(\alpha \sigma \right)\right]$ and $\ln\dot{\varepsilon }$, as well as between $\ln\left[\sinh\left(\alpha \sigma \right)\right]$ and 1/*T*, were obtained, as shown in Figure 5. From the slopes of these plots, the *Q* value was determined to be 405.559 kJ/mol.

Considering the influence of temperature and strain rate on flow stress, the Zener-Hollomon (*Z*) parameter is introduced as follows:

(7) $$Z=\dot{\varepsilon }exp\left(Q/RT\right)$$

Substituting Equation (1) into Equation (7) yields the following relationship (Equation (8)):

(8) $$Z=A{\left[\sinh\left(\alpha \sigma \right)\right]}^{n}$$

**Figure 6:**
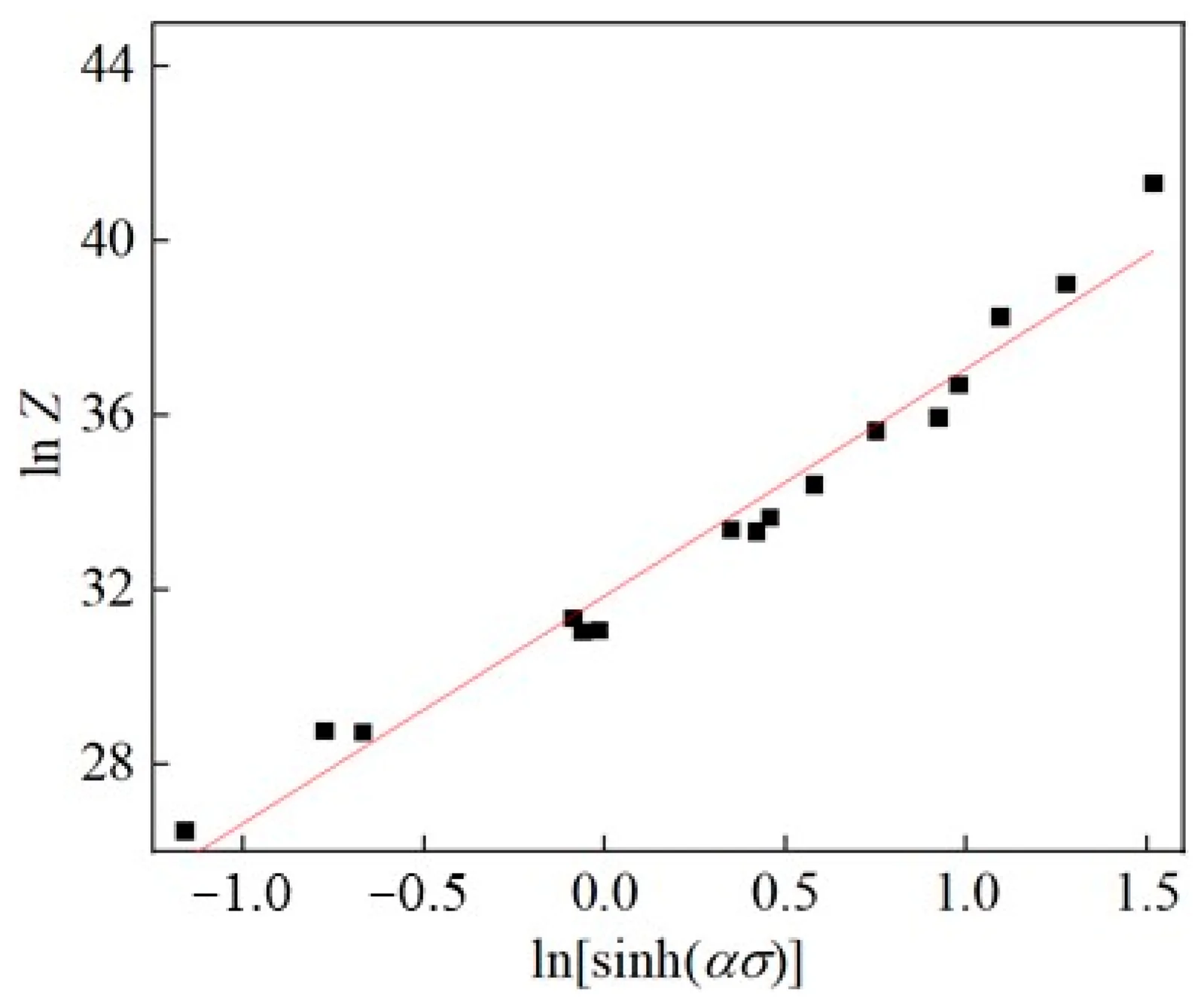
Relationship between $\ln\left[\sinh\left(\alpha \sigma \right)\right]$ and ln*Z*.

Figure 6 shows the relationship between *Z* and $\ln\left[\sinh\left(\alpha \sigma \right)\right]$ at a strain of 0.5. It can be calculated that *n* = 5.2128 and ln*A* = 31.86. A linear regression of the data points yields a slope of *n* = 5.2128 and an intercept of ln*A* = 31.86.

According to Equation (1), the expression of flow stress can be obtained, as shown in Equation (9),

(9) $$\sigma =\frac{1}{\alpha }\ln\left\{{\left[\frac{\dot{\varepsilon }exp\left(\frac{Q}{RT}\right)}{A}\right]}^{\frac{1}{n}}+{\left[{\left(\frac{\dot{\varepsilon }exp\left(\frac{Q}{RT}\right)}{A}\right)}^{\frac{2}{n}}+1\right]}^{\frac{1}{2}}\right\}$$

**Figure 7:**
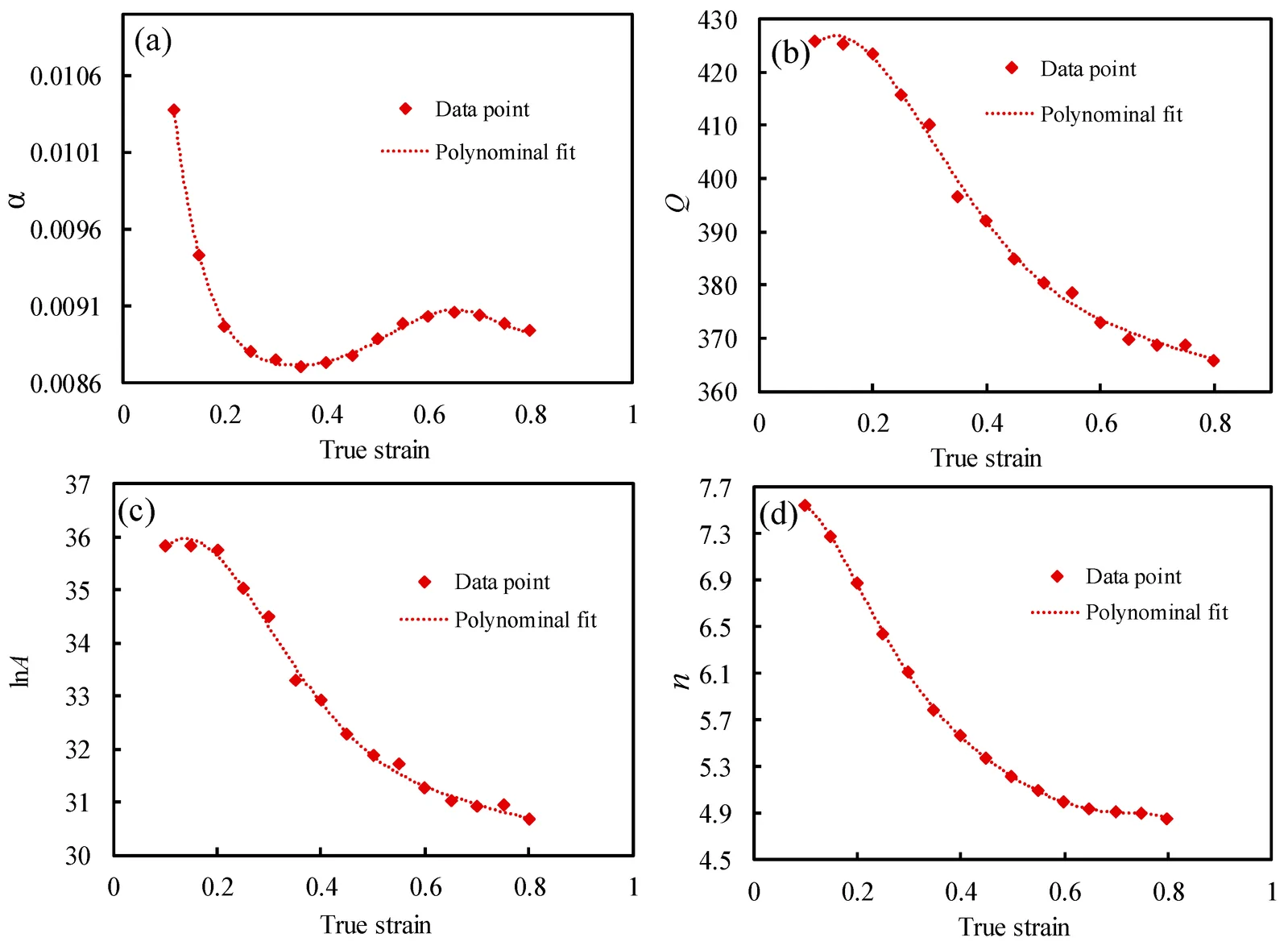
The variations of α, *Q*, *n*, lnA with strain. (**a**) Relationship between α and strain. (**b**) Relationship between *Q* and strain. (**c**) Relationship between lnA and strain. (**d**) Relationship between *n* and strain.

Following the same procedure, the values of *α*, *Q*, *n*, ln *A* were calculated at strains ranging from 0.1 to 0.8, and the results are presented in Figure 7. To characterize the strain dependence of these material constants, polynomial fitting was performed, and the resulting relationships are expressed in Equation (10). Based on these strain-compensated parameters, a comprehensive constitutive model was established to predict the flow stress of the 022Cr12Ni10MoTi alloy as a function of strain, strain rate, and deformation temperature.

(10) $$\left\{\begin{array}{c} \alpha =0.7019\varepsilon^{6}-2.0249\varepsilon^{5}+2.3525\varepsilon^{4}-1.4254\varepsilon^{3}+0.4847\varepsilon^{2}-0.089\varepsilon +0.0156\\ Q=2274.4\varepsilon^{6}-4238.9\varepsilon^{5}+803.32\varepsilon^{4}+3026.4\varepsilon^{3}-2309.6\varepsilon^{2}+454.67\varepsilon +400.03\\ n=-436.8\varepsilon^{6}+1257.9\varepsilon^{5}-1449.4\varepsilon^{4}+844.41\varepsilon^{3}-249.96\varepsilon^{2}+27.325\varepsilon +6.5917\\ \ln A=125.32\varepsilon^{6}-147.64\varepsilon^{5}-208.95\varepsilon^{4}+446.42\varepsilon^{3}-265.92\varepsilon^{2}+50.594\varepsilon +32.957 \end{array}\right.$$

**Figure 8:**
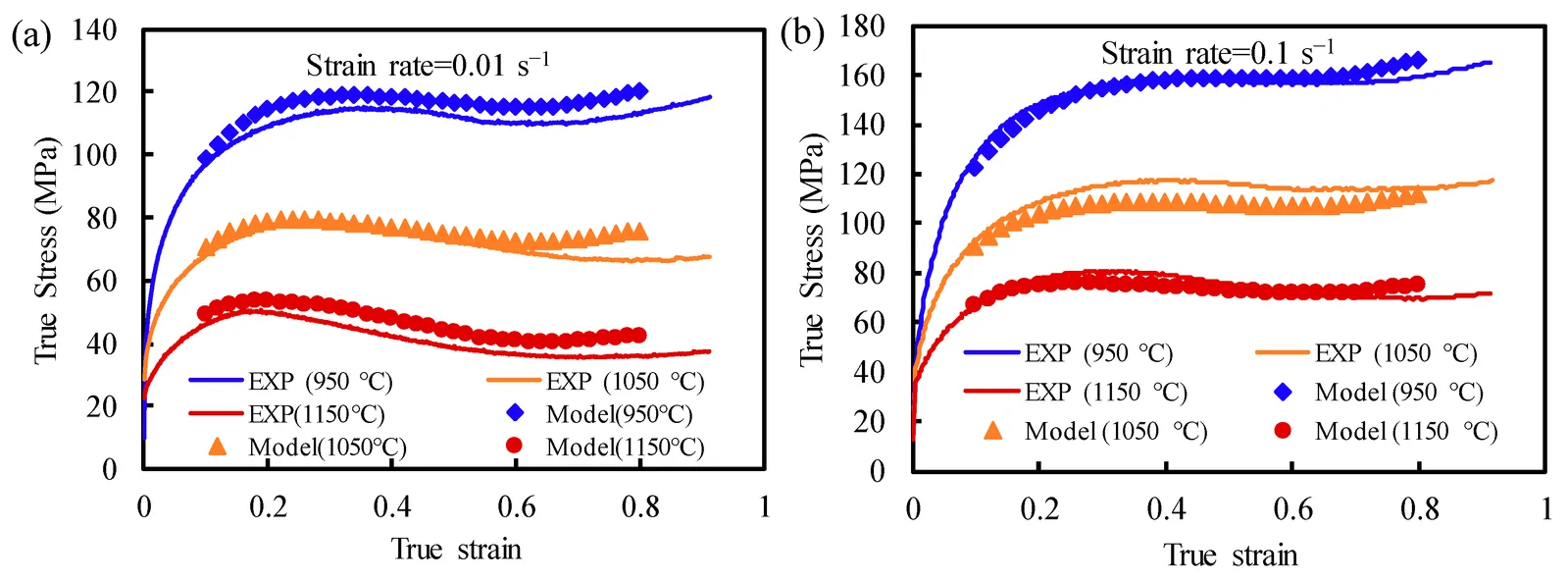

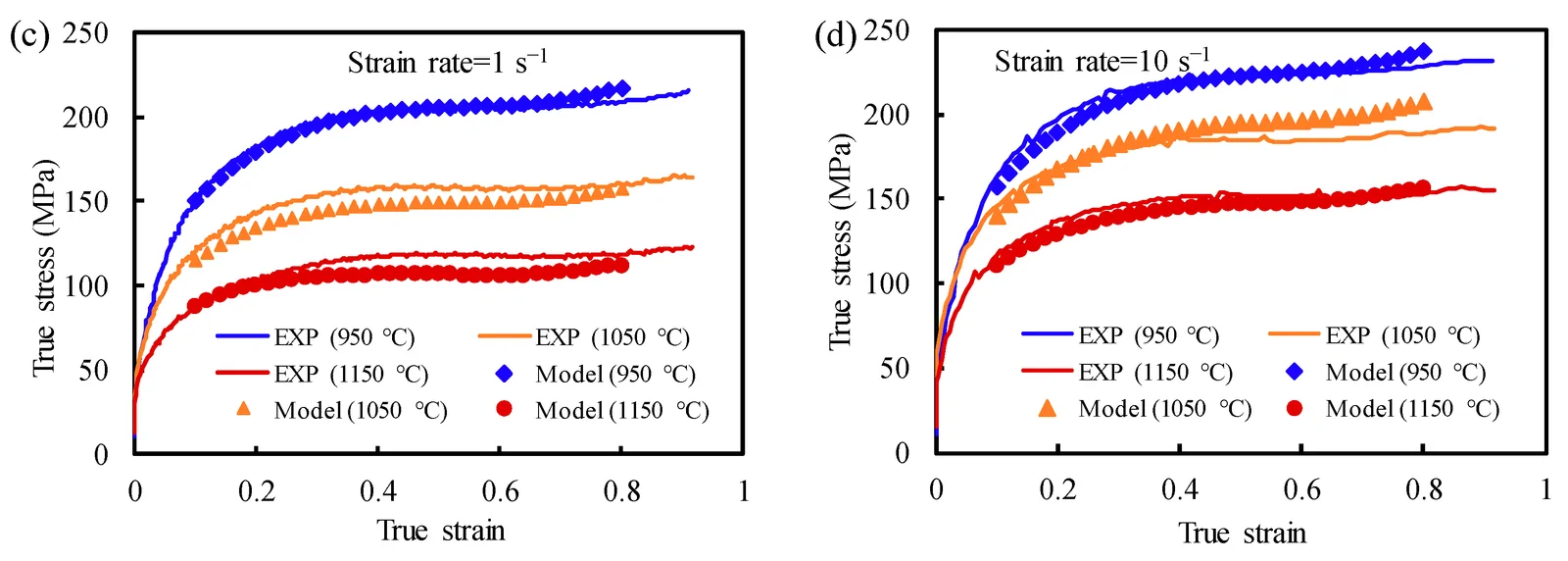
Comparison of the experimental and strain-compensated predicted flow-stress at different strain rates. (**a**) Strain rate = 0.01 s^−1^. (**b**) Strain rate = 0.1 s^−1^. (**c**) Strain rate = 1 s^−1^. (**d**) Strain rate = 10 s^−1^.

Based on the stress–strain curves obtained under various strain rates and temperatures, the proposed constitutive model was evaluated and subsequently validated against experimental data, as shown in Figure 8. It can be seen that the maximum error occurs at a temperature of 1050 °C and a strain rate of 0.01 s^−1^. Under this condition, the experimental value is 67 MPa and the predicted value is 74.5 MPa; thus, the maximum error is approximately 11%. The predicted results are in good agreement with the experimental results, indicating that the established constitutive equations are capable of reliably predicting the mechanical behavior of 022Cr12Ni10MoTi alloy during hot deformation.

### 4.2. Processing Map

According to the dynamic material modeling (DMM) theory, a material undergoing hot deformation behaves as a nonlinear power dissipator. During this process, the total power absorbed per unit volume, denoted as *P*, is partitioned into two complementary components: the power dissipation capacity *G*, which accounts for the energy dissipated by plastic work, and the power dissipation co-content *J*, which is associated with the energy dissipated through microstructural evolution mechanisms. The relationship between these components is given by the following expression [29]:

(11) $$P=\sigma \times \dot{\varepsilon }=G+J=\int_{0}^{\dot{\varepsilon }}\sigma \times d\dot{\varepsilon }+\int_{0}^{\sigma }\dot{\varepsilon }\times d\sigma$$

During hot deformation, the majority of the absorbed power is dissipated in the form of heat, while a relatively smaller fraction is consumed by microstructural evolution. The partitioning of the total power between the dissipator content *G* and the dissipator co-content *J* is governed by the strain rate sensitivity index *m*, which is defined as follows:

(12) $$m=\frac{\partial J}{\partial G}=\frac{\partial \ln\sigma }{\partial \ln\dot{\varepsilon }}$$

In general, the strain rate sensitivity index *m* varies nonlinearly with deformation temperature and strain rate. Consequently, for a nonlinear power dissipation process, the power dissipation efficiency *η* can be expressed as follows:

(13) $$\eta =\frac{J}{J_{max}}=\frac{2m}{1+m}$$

where *J*_max_ denotes the maximum value of the dissipation co-content *J* attainable under ideal linear dissipation conditions (i.e., when m = 1). The power dissipation efficiency factor *η* represents the ratio of the energy dissipated by microstructural evolution during material forming, *J*, to the theoretical maximum max *J*_max_ under linear dissipation.

A higher power dissipation efficiency is indicative of favorable conditions for hot workability; however, not all regions exhibiting high dissipation efficiency are suitable for hot processing. Therefore, it is essential to first identify the flow instability regimes of the material. Based on the principle of maximum entropy production rate for large-strain plastic deformation, the criterion for flow instability is given by Equation (14):

(14) $$\zeta \left(\dot{\varepsilon }\right)=\frac{\partial \ln\left(\frac{m}{m+1}\right)}{\partial \ln\dot{\varepsilon }}+m<0$$

where the parameter $\zeta \left(\dot{\varepsilon }\right)$ assumes negative values are identified as flow instability domains. By applying Equation (13), the power dissipation map can be constructed, while Equation (14) enables the development of the processing instability map. Overlaying the power dissipation map with the instability map yields the processing map for hot deformation.

**Figure 9:**
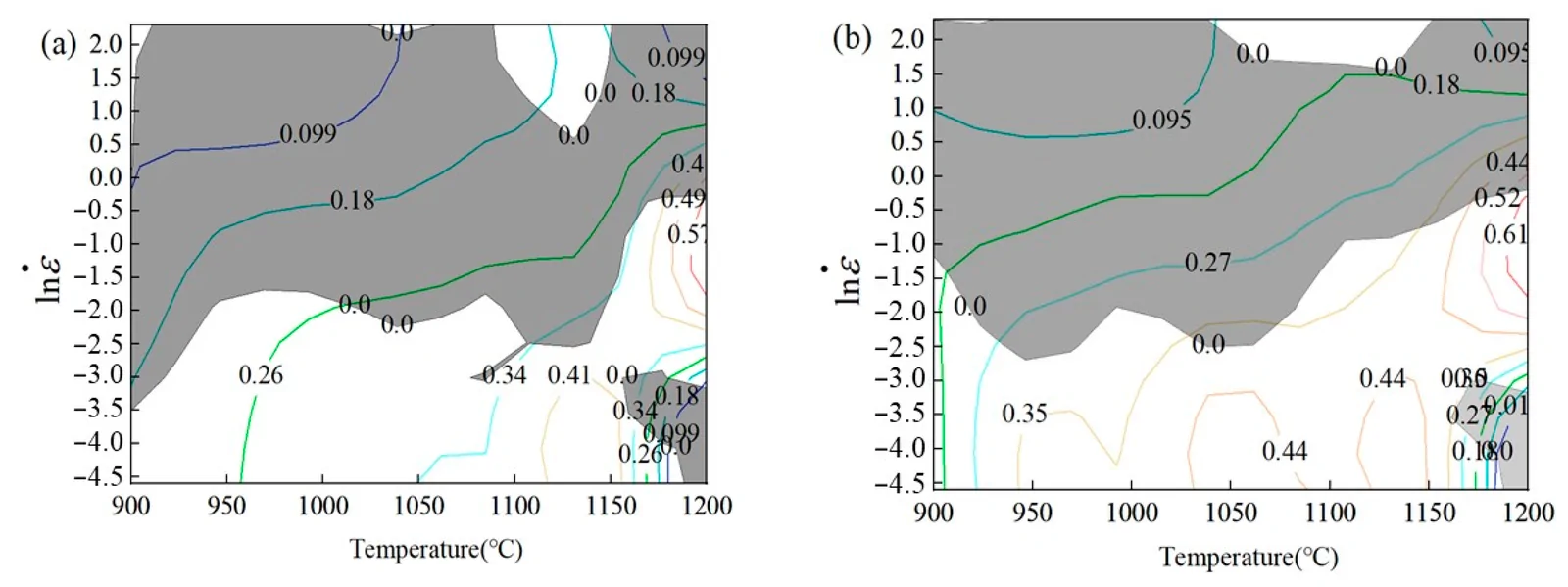
Processing maps of 022Cr12Ni10MoTi alloy. (**a**) Strain = 0.4. (**b**) Strain = 0.8.

The processing maps of the 022Cr12Ni10MoTi alloy were established within the temperature range of 900–1200 °C and strain rate range of 0.01–10 s^−1^, as shown in Figure 9. This temperature interval covers the full forging process window of the 022Cr12Ni10MoTi alloy from initial forging to finish forging. It can characterize the evolution behaviors, including the re-solution of precipitates and austenite grain coarsening at high temperatures, as well as dynamic precipitation, dynamic recovery, and recrystallization at relatively low temperatures. The adopted strain rate range covers various industrial forming rates such as isothermal slow forging, conventional die forging, and high-speed hammer forging, enabling a sufficiently wide range of Zener-Hollomon parameters. Meanwhile, it avoids experimental errors induced by static microstructure evolution at extremely low strain rates, as well as indenter inertia and adiabatic temperature rise at excessively high strain rates.

The results in Figure 9 show that the power dissipation efficiency increases with increasing deformation temperature and decreasing strain rate. However, an exception exists within the temperature range of 1150 °C to 1200 °C and strain rate range of 0.01–0.1 s^−1^, where this trend is reversed. The instability regions are indicated by the shaded areas in Figure 9. It can be observed that flow instability mainly occurs at high strain rates (i.e., greater than 0.1 s^−1^). In addition, instability also appears in the region with high temperature and low strain rate, specifically at 1150–1200 °C and 0.01–0.1 s^−1^. The power dissipation efficiency within these instability zones is relatively low, generally below 0.35. Accordingly, it can be concluded that the optimal hot deformation processing conditions for this alloy are a temperature of 900–1150 °C and a strain rate of 0.01–0.1 s^−1^.

**Figure 10:**
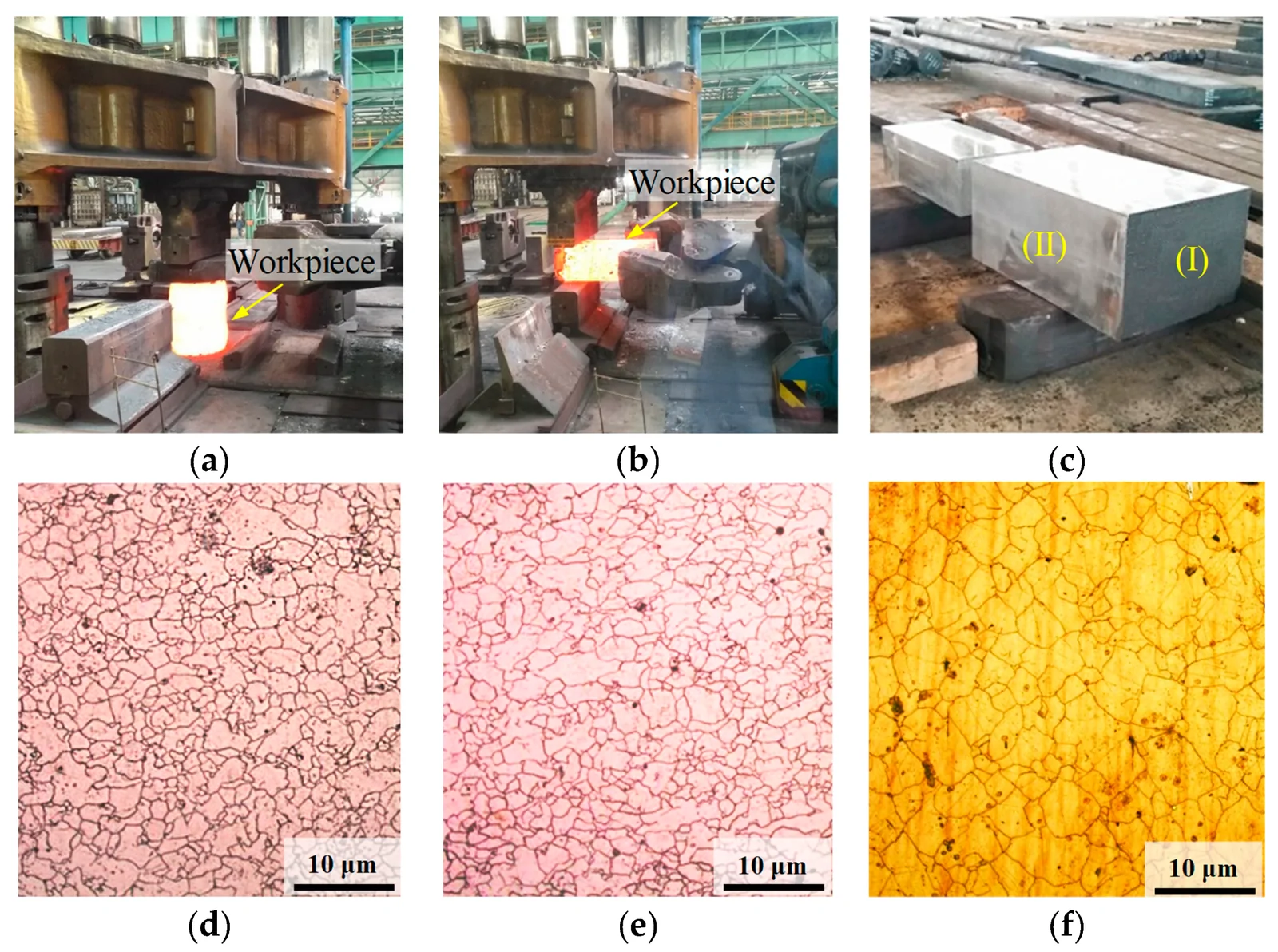
Forging process and microstructure of 022Cr12Ni10MoTi alloy. (**a**) Upsetting process. (**b**) Drawing-out process. (**c**) Forged component. (**d**) Microstructure at I. (**e**) Microstructure at II. (**f**) Central microstructure.

Based on the processing maps obtained above, hot forging trials were conducted on large-scale 022Cr12Ni10MoTi alloy components with dimensions of 600 mm × 450 mm × 1000 mm, as shown in Figure 10. The initial forging temperature was set at 1140 °C, within the identified optimal processing window. The material was subjected to repeated hot upsetting (Figure 10a) and drawing (Figure 10b) processes at elevated temperatures to refine the microstructure and enhance the mechanical properties. The final dimensions of the forged specimen are presented in Figure 10c. Microstructural observations were carried out at three representative locations: the transverse half-section, the longitudinal half-section, and the central interior region, with the corresponding microstructures shown in Figure 10d–f, respectively. The grain size distributions at these locations were quantitatively analyzed. At location I (transverse half-section), the distribution was 7.0 μm (40%) + 5.5 μm (40%) + 4.0 μm (20%); at location II (longitudinal half-section), it was 6.0 μm (40%) + 4.0 μm (40%) + 3.0 μm (20%); and at the central interior region, it was 7.0 μm (68%) + 4.0 μm (30%) + others (2%). Overall, the grain size across the entire forging ranged from 3 μm to 7 μm, indicating that the grains were effectively refined. Moreover, the grain size distributions at the three examined locations within the large-scale specimen were found to be relatively uniform, demonstrating the feasibility of the identified processing window for industrial-scale applications.

## 5. Conclusions

This study systematically investigated the hot deformation behavior of 022Cr12Ni10MoTi alloy. The evolution of mechanical properties and microstructure during hot deformation was comprehensively characterized, a constitutive model was developed, and processing maps were constructed. The main conclusions are summarized as follows:

1. The flow stress–strain curves of the 022Cr12Ni10MoTi alloy during hot deformation were obtained, revealing pronounced thermal softening and strain rate hardening effects. Microstructural observations confirmed the occurrence of dynamic recovery and recrystallization. Lower strain rates and higher temperatures were found to promote recovery and recrystallization during hot deformation. When the deformation temperature exceeded 1200 °C, noticeable grain coarsening of the recrystallized grains was observed.
2. The constitutive parameters of the 022Cr12Ni10MoTi alloy were determined based on the high-temperature flow stress–strain data, and a constitutive model was established to describe its hot deformation behavior. The predictions of the developed constitutive model show good agreement with the experimental results, with a relative error of less than 11%, demonstrating that the proposed constitutive model can accurately characterize the mechanical response of the alloy during hot deformation.
3. Processing maps for the 022Cr12Ni10MoTi alloy were constructed based on the developed constitutive model. The maps reveal that the optimal hot-deformation conditions lie within the temperature range of 900 –1150 °C and the strain rate range of 0.01–0.1 s^−1^. Guided by these results, hot forging trials were carried out on large-scale specimens. The microstructures obtained after forging exhibited significant grain refinement and good uniformity, with grain sizes ranging from approximately 3 μm to 7 μm.

## Figures and Tables

**Table 1:** Chemical composition of 022Cr12Ni10MoTi alloy (wt %).

Chemical Composition	C	Si	Mn	S	P	Cr	Ni	Mo	Al	Ti
Indicator requirements	≤0.03	≤0.15	≤0.15	≤0.008	≤0.01	11.50~12.50	9.0~10.3	0.5~0.8	≤0.2	≤0.25

## Data Availability

The original contributions presented in this study are included in the article. Further inquiries can be directed to the corresponding author.
